# The first coordination compound of 6-fluoro­nicotinate: the crystal structure of a one-dimensional nickel(II) coordination polymer containing the mixed ligands 6-fluoro­nicotinate and 4,4′-bi­pyridine

**DOI:** 10.1107/S2056989020003023

**Published:** 2020-03-10

**Authors:** Nives Politeo, Mateja Pisačić, Marijana Đaković, Vesna Sokol, Boris-Marko Kukovec

**Affiliations:** a Department of Physical Chemistry, Faculty of Chemistry and Technology, University of Split, Ruđera Boškovića 35, HR-21000 Split, Croatia; b Department of Chemistry, Faculty of Science, University of Zagreb, Horvatovac, 102a, HR-10000 Zagreb, Croatia

**Keywords:** crystal structure, nickel(II), 6-fluoro­nicotinic acid, 4,4′-bi­pyridine, coordination polymer, hydrogen-bond motif

## Abstract

A one-dimensional nickel(II) coordination polymer with the mixed ligands 6-fluoro­nicotinate (6-Fnic) and 4,4′-bi­pyridine (4,4′-bpy), namely, *catena*-poly[[di­aqua­bis­(6-fluoro­pyridine-3-carboxyl­ato-κ*O*)nickel(II)]-μ-4,4′-bi­pyri­dine-κ^2^ *N*:*N*′] trihydrate], {[Ni(6-Fnic)_2_(4,4′-bpy)(H_2_O)_2_]·3H_2_O}_*n*_, (**1**), was prepared by the reaction of nickel(II) sulfate hepta­hydrate, 6-fluoro­nicotinic acid (C_6_H_4_FNO_2_) and 4,4′-bi­pyridine (C_10_H_8_N_2_) in a mixture of water and ethanol. The nickel(II) ion in **1** is octa­hedrally coordinated by the O atoms of two water mol­ecules, two O atoms from *O*-monodentate 6-fluoro­nicotinate ligands and two N atoms from bridging 4,4′-bi­pyridine ligands, forming a *trans* isomer. The bridging 4,4′-bi­pyridine ligands connect symmetry-related nickel(II) ions into infinite one-dimensional polymeric chains running in the [10] direction. In the extended structure of **1**, the polymeric chains and lattice water mol­ecules are connected into a three-dimensional hydrogen-bonded network *via* strong O—H⋯O and O—H⋯N hydrogen bonds, leading to the formation of distinct hydrogen-bond ring motifs: octa­meric *R*_8_^8^(24) and hexa­meric *R*_8_^6^(16) loops.

## Abstract 2

A one-dimensional nickel(II) coordination polymer with the mixed ligands 6-fluoro­nicotinate (6-Fnic) and 4,4′-bi­pyridine (4,4′-bpy), namely, [Ni(H_2_O)_2_(6-Fnic)_2_(4,4′-bpy)·3H_2_O]_*n*_, (**1**), was prepared. The nickel(II) ion in **1** is octa­hedrally coordinated by the O atoms of two water mol­ecules, two O atoms from *O*-monodentate 6-fluoro­nicotinate ligands and two N atoms from bridging 4,4′-bi­pyridine ligands.

## Chemical context   

The design of coordination polymers relies on the concepts of crystal engineering (Desiraju, 2007 ▸, 2013 ▸) and has become a prominent field of research in recent years for many reasons including the functional properties shown by coordination polymers, their aesthetics and many possible applications such as catalysis, gas storage and separation, magnetism, luminescence and mol­ecular sensing (Mueller *et al.*, 2006 ▸; Bosch *et al.*, 2017 ▸; Zhang *et al.*, 2015 ▸; Zeng *et al.*, 2014 ▸, 2016 ▸; Douvali *et al.*, 2015 ▸; Xu *et al.*, 2017 ▸; Zhou *et al.*, 2017 ▸). The multifunctionality of the organic ligands used as building blocks in the assembly of coordination polymers is reflected in the position and coordination ability of their donor atoms and/or groups and is the main factor in the design of unusual and unexpected architectures with novel topologies and properties. The main challenge is to control the formation of a coordination polymer with the desired mol­ecular and crystal structure, which is particularly affected by the experimental conditions such as the choice of solvents, starting metal salts, additional ligands, temperature, hydro­thermal conditions, pH value (Li *et al.*, 2016 ▸; Zhou *et al.*, 2016 ▸; Gu *et al.*, 2016 ▸).

Various aromatic carb­oxy­lic acids with additional functional groups have often been used in the construction of coordination polymers because of the variety of their coordination modes (often unpredictable) and their potential for forming supra­molecular inter­actions (Gu *et al.*, 2016 ▸, 2017 ▸, 2018 ▸; Wang *et al.*, 2016 ▸; Zhang *et al.*, 2019 ▸). Fluorine-substituted aromatic carb­oxy­lic acids are good candidates for the design of functional coordination polymers showing higher thermal stability as well as stability towards oxidation (Peikert *et al.*, 2015 ▸; Yuan *et al.*, 2016 ▸).

Although metal complexes with nicotinate have been well-studied and documented [almost 900 crystal structures in the Cambridge Structural Database (CSD, Version 5.40, searched January 2020; Groom *et al.*, 2016 ▸)], metal complexes of its fluorinated analogues (*e.g*. 5-fluoro­nicotinate) have been much less studied (around 30 crystal structures in the CSD). On the other hand, no metal complexes of other fluorinated analogues of nicotinate (*e.g*. 2-fluoro­nicotinate, 4-fluoro­nicotinate, 6-fluoro­nicotinate) have been reported so far.

Our goal was to prepare nickel(II) coordination polymers as the nickel(II) ion is relatively abundant, with a large ionic radius and defined stereochemistry, showing a high ligand-field stabilization energy, which enables the formation of nickel(II) coordination polymers with diverse topologies and high stabilities (Liu *et al.*, 2019 ▸). We opted for nickel(II) coordination polymers with mixed ligands: 6-fluoro­nicotinate (6-Fnic) as the main ligand and 4,4′-bi­pyridine (4,4′-bpy), a well-established, bridging N-donor ligand, frequently used in the design of nickel(II) coordination polymers, as the supporting ligand.
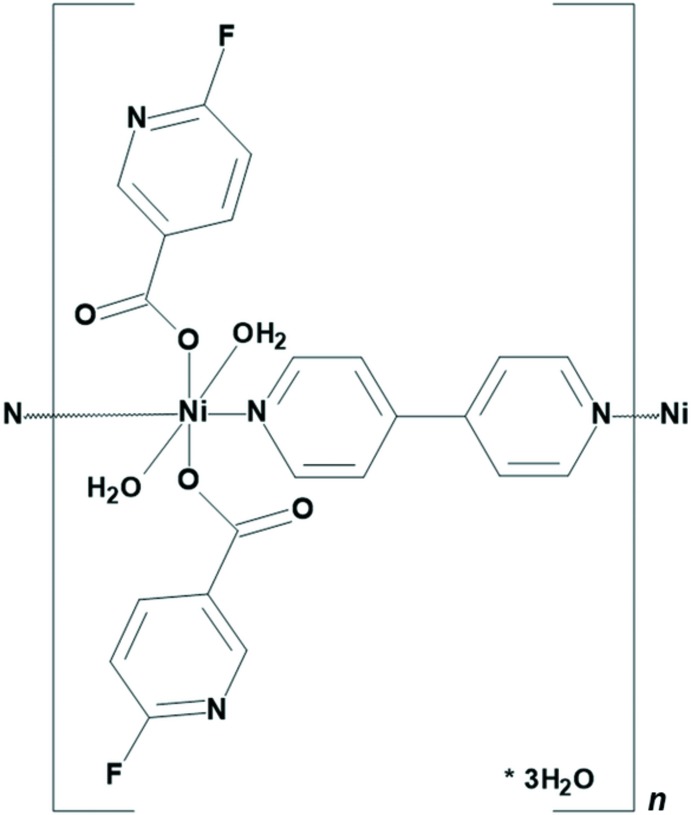


In this work, we report the synthesis and characterization of the first metal complex with 6-fluoro­nicotinate – the one-dimensional nickel(II) coordination polymer {[Ni(6-Fnic)_2_(4,4′-bpy)(H_2_O)_2_]·3H_2_O} (**1**). The synthesis was carried out in a mixture of water and ethanol in the hope that the coord­inated water mol­ecules would complete the coordination sphere around the nickel(II) ion and participate in the formation of various hydrogen-bond motifs within the hydrogen-bonded framework, along with the anti­cipated lattice water mol­ecules. Furthermore, we wanted to explore the effect of the probable weak inter­molecular inter­actions involving the aromatic F atoms (for example C—H⋯F inter­actions) on the assembly of the polymeric chains in the crystal packing.

## Structural commentary   

**Figure 1:**
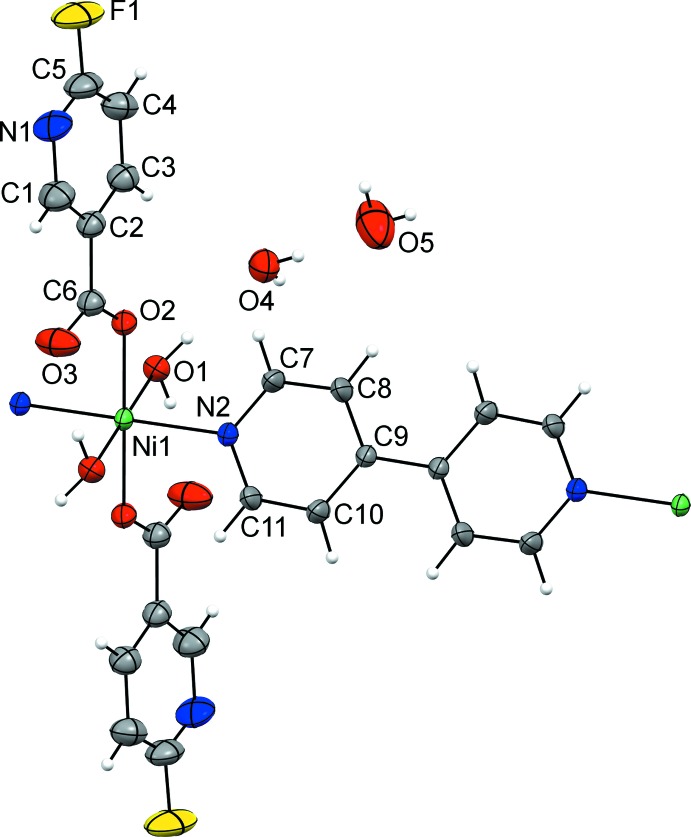
The repeating polymeric unit of **1**, showing the atomic numbering scheme of the asymmetric unit. The displacement ellipsoids are drawn at the 40% probability level.

**Figure 2:**
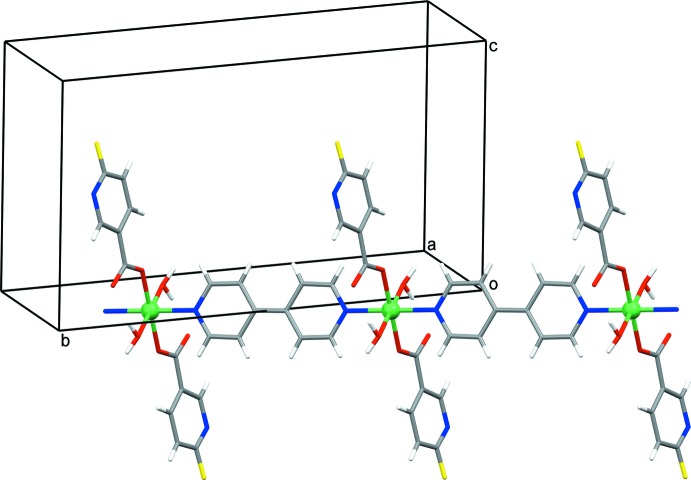
An infinite one-dimensional polymeric chain of **1** showing the connectivity.

As the nickel(II) ion and the lattice water mol­ecule (atom O4) are situated on an inversion center and a twofold axis, respectively, the asymmetric unit of **1** consists of one half of a nickel(II) ion, one coordinated water mol­ecule, one fluoro­nicotinate ligand, one half of a 4,4′-bi­pyridine ligand and one and a half lattice water mol­ecules. The nickel(II) ion is octa­hedrally coordinated by two 6-fluoro­nicotinate O atoms (O2 and O2^i^) and by two 4,4′-bi­pyridine N atoms (N2 and N2^i^) in the equatorial position, whilst two water mol­ecule O atoms (O1 and O1^i^) are bound in the axial positions [symmetry code: (i) −*x* + 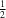, −*y* + 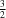, −*z*]. In this way, a *trans* isomer is formed (N2^i^—Ni1—N2 = 180°) (Fig. 1 ▸). The 6-fluoro­nicotinate ligands are bound to the nickel(II) ion *via* their carboxyl­ate O atoms in an *O*-monodentate fashion, whilst the 4,4′-bi­pyridine ligands act as bridge and, thus, connect the symmetry-related nickel(II) ions into an infinite one-dimensional polymeric chain extending in the [1
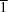
0] direction (Fig. 2 ▸). There are three lattice water mol­ecules per repeating polymeric unit, {[Ni(6-Fnic)_2_(4,4′-bpy)(H_2_O)_2_]·3H_2_O}.

The octa­hedral coordination environment around the nickel(II) ion is slightly distorted, as indicated by the angles for the *cis* pairs of the ligating atoms [89.65 (6)–90.87 (6)°]. The Ni1—O1 bond length [2.1067 (16) Å] is somewhat longer than the Ni1—O2 and Ni1—N2 bond lengths [2.0553 (13) and 2.0570 (16) Å, respectively], which is in agreement with the fact that the water mol­ecules are bound in the axial positions of the octa­hedron. The Ni—O_c_ (c = carboxyl­ate) bond lengths in **1** are comparable to those seen in the related nickel(II) complexes with 6-chloro­nicotinate (Xia *et al.*, 2012 ▸), 5-fluoro­nicotinate (Cui *et al.*, 2015 ▸), mixed 5-fluoro­nicotinate and 2,2′-bi­imidazole ligands (Li *et al.*, 2019 ▸), mixed 5-bromo­nicotinate and 1,1′-(5-methyl-1,3-phenyl­ene)bis­(1*H*-imidazole) ligands (Lv *et al.*, 2016 ▸), 5-chloro­nicotinate (Chen *et al.*, 2019 ▸) and mixed 5-chloro­nicotinate and 2,2′-bi­imidazole ligands (Chen *et al.*, 2019 ▸). The Ni—N bond lengths are in agreement with those reported for nickel(II) complexes containing bridging 4,4′-bi­pyridine ligands (Groom *et al.*, 2016 ▸).

The 4,4′-bypyridine ring is not coplanar with the coordinated water mol­ecule atom O1, but it is rotated slightly (approximately 4°) about the Ni1—N2 bond, as is evident from the torsion angles Ni1—N2—C7—C8 [176.35 (19)°] and Ni1—N2—C11—C10 [−176.03 (18)°]. The values of these torsion angles ought to be 180° in the case of coplanarity of the 4,4′-bi­pyridine ring and the O1 atom of the coordinated water mol­ecule.

## Supra­molecular features   

**Figure 3:**
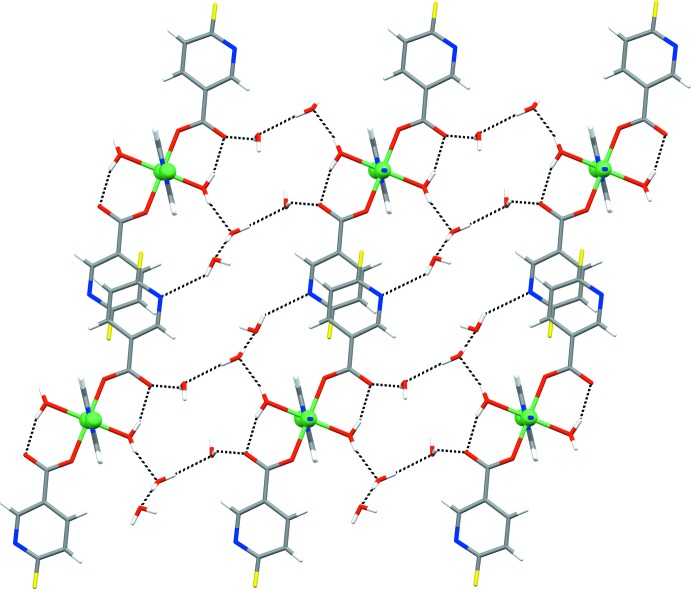
A fragment of the infinite two-dimensional hydrogen-bonded network of **1** viewed along the [1
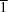
0] direction. The polymeric chains, represented as monomeric mol­ecules in this projection, and lattice water mol­ecules are connected by O—H⋯O and O—H⋯N hydrogen bonds (represented by dotted lines) within the network. The polymeric chains are additionally assembled by π–π inter­actions between symmetry-related 6-fluoro­nicotinate pyridine rings.

The extended structure of **1** mainly features strong O—H⋯O and O—H⋯N hydrogen bonds (Table 1 ▸) and π–π inter­actions [*Cg*1⋯*Cg*1(−*x* + 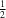, −*y* + 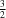, −*z* + 1) = 3.8148 (16) Å; dihedral angle between the planes = 0.00 (14)°; slippage = 1.792 Å and *Cg*1⋯*Cg*2(*x* + 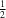, 2 − *y*, 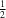 + *z*) = 3.8798 (16) Å; dihedral angle between the planes = 11.68 (13)°; slippage = 1.917 Å; *Cg*1 and *Cg*2 are the centroids of the 6-fluoro­nicotinate pyridine (N1/C1–C5) and 4,4′-bi­pyridine (N2/C7–C11) rings, respectively]. The strong hydrogen bonds link the polymeric chains and the lattice water mol­ecules into an infinite three-dimensional network. The structure can be better analysed if viewed down the [1
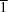
0] direction (the direction along which the polymeric chain is running). In that projection, the polymeric chains can be regarded as monomeric mol­ecules that are inter­connected with lattice water mol­ecules into an infinite two-dimensional hydrogen-bonded network (Fig. 3 ▸). While being exclusively hydrogen-bonded to lattice water mol­ecules, the polymeric chains are additionally directly assembled by π–π inter­actions between symmetry-related 6-fluoro­nicotinate pyridine rings [*Cg*1⋯*Cg*1].

**Figure 4:**
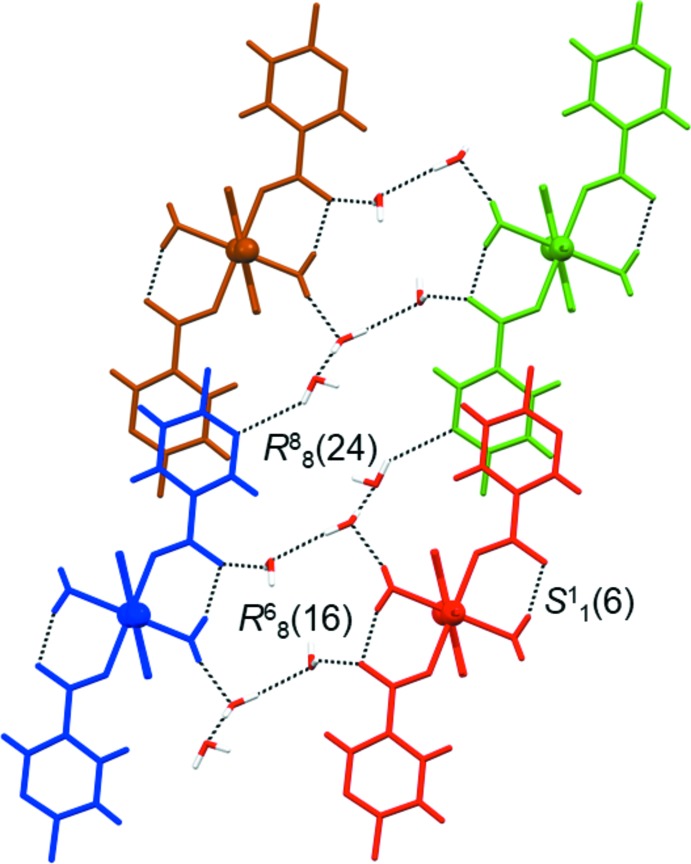
The distinctive hydrogen-bonded ring motifs (represented by the dotted lines) found within the two-dimensional network of **1** viewed along the [1
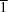
0] direction, *viz*. octa­meric 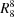
(24), hexa­meric 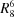
(16) and intra­molecular 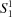
(6) motifs. The various symmetry-related polymeric chains (represented as momomeric mol­ecules in this projection) are shown in brown, green, blue and red (see text).

There are some distinctive hydrogen-bonded ring motifs within the two-dimensional network of **1** (Fig. 4 ▸). The octa­meric 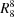
(24) motif is formed between six lattice water mol­ecules and two symmetry-related polymeric chains (indicated in blue and green), which are linked *via* two 6-fluoro­nicotinate pyridine N atoms and two carboxyl­ate O atoms. The hexa­meric 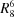
(16) motif is formed between four lattice water mol­ecules and two symmetry-related polymeric chains (indicated in blue and red), which are linked *via* two coordinated water mol­ecules and two carboxyl­ate O atoms, while the intra­molecular 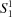
(6) motif is formed within the polymeric chain (indicated in red) *via* a coordinated water mol­ecule and a carboxyl­ate O atom (Fig. 4 ▸). Both coordinated and lattice water mol­ecules participate in the formation of motifs as single- and double-proton donors [coordinated water mol­ecules as single-proton donors in the 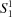
(6) motif and double-proton donors in the 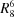
(16) motif; lattice water mol­ecules as single-proton donors in the 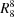
(24) and 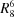
(16) motifs and double-proton donors in the 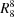
(24) motif]. The 6-fluoro­nicotinate pyridine N atoms act as single-proton acceptors exclusively, while carboxyl­ate O atoms act as both single- and double-proton acceptors [single in the 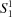
(6) and 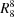
(24) motifs and double in the 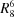
(16) motif].

Although there are many reported nickel(II) coordination polymers containing bridging 4,4′-bi­pyridine and pyridinedi­carboxyl­ate ligands, there are only two structurally similar one-dimensional nickel(II) polymers with 4,4′-bi­pyridine and pyridine­carboxyl­ate (*i.e.* picolinate; Li *et al.*, 2009 ▸) or fluorinated benzoate (2,6-di­fluoro­benzoate; Yuan *et al.*, 2016 ▸) ligands. The polymeric chains are assembled with lattice water mol­ecules in the crystal packing of the picolinate polymer (Li *et al.*, 2009 ▸) or with the solvated ethanol mol­ecules in the crystal packing of the 2,6-di­fluoro­benzoate polymer (Yuan *et al.*, 2016 ▸). The discussed hydrogen-bond motifs in **1** are completely different from those observed in the crystal packings of these similar polymers, except for the intra­molecular 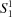
(6) motif, which is also present in the packing of the 2,6-di­fluoro­benzoate polymer (Yuan *et al.*, 2016 ▸). The reason for the different hydrogen-bond motifs may be due to the different arrangement of the lattice water mol­ecules (primarily connected to each other into a layered network and not extensively connected to the polymeric chains) in the packing of the picolinate polymer (Li *et al.*, 2009 ▸), and the fact that the ethanol O atoms are solely proton acceptors (not being able to participate in extensive hydrogen bonding as water mol­ecules) in the packing of the 2,6-di­fluoro­benzoate polymer (Yuan *et al.*, 2016 ▸).

Unfortunately, there are no weak inter­molecular inter­actions involving the aromatic F atoms; we hoped these inter­actions could have an effect on the supra­molecular assembly of the polymeric chains in **1**. The reason for the lack of such inter­actions may be the extensive hydrogen bonding, comprising strong O—H⋯O and O—H⋯N hydrogen bonds, that hinders weak C—H⋯F supra­molecular inter­actions. Indeed, the crystallization from an aqueous solution enabled the participation of the lattice water mol­ecules in the extended structure of **1**, enhancing the number of O—H⋯O and O—H⋯N hydrogen bonds in the hydrogen-bonded network and leading to the formation of the anti­cipated hydrogen-bond motifs.

## PXRD and thermal analysis   

**Figure 5:**
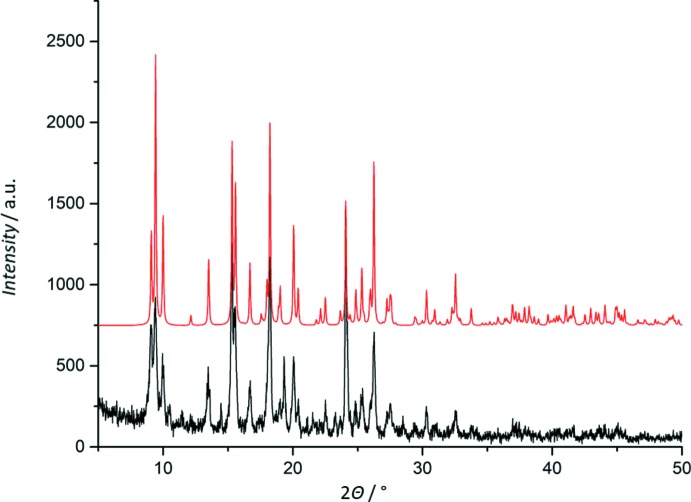
Experimental (bottom) and calculated (top) PXRD spectra of **1**.

The PXRD analysis was used to confirm the bulk content of **1** (Fig. 5 ▸). The experimental and calculated PXRD spectra of **1** are in very good agreement.

The thermal stability of **1**, as determined from the TG curve, is only up to 40°C (Fig. S1 in the supporting information). Both the coordinated (two) and lattice (three) water mol­ecules were released in the same step (observed mass loss 14.5%, calculated 15.4%). The two small endothermic peaks in the DSC curve (63 and 115°C) suggest that the process of the water evolution is not straightforward and that the water mol­ecules are differently bound in **1** (coordinated *vs* lattice). Indeed, the polymeric chains and lattice water mol­ecules are assembled into a hydrogen-bonded three-dimensional structure (see *Supra­molecular features*). It is therefore not surprising that the release of some water mol­ecules affects the whole hydrogen-bonded structure and leads to its complete collapse in a single, not well-resolved thermal step. The thermal decomposition of **1** continues in a broad step (observed mass loss 56.7%) in the wide temperature range of 150–570°C (without any well-defined peaks in the DSC curve), which probably corresponds to the complete degradation of **1**. The remaining residue at 600°C is most probably NiO.

## Materials and methods   

All chemicals for the synthesis were purchased from commercial sources (Merck, ChemPUR) and used as received without further purification. The IR spectrum was obtained in the range 4000–400 cm^−1^ on a Perkin–Elmer Spectrum Two^TM^ FTIR spectrometer in the ATR mode. The PXRD trace was recorded on a Philips PW 1850 diffractometer, Cu *K*α radiation, voltage 40 kV, current 40 mA, in the angle range 5–50° (2*θ*) with a step size of 0.02°. Simultaneous TGA/DSC measurements were performed at a heating rate of 10°C min^−1^ in the temperature range 25–600°C, under a nitro­gen flow of 50 mL min^−1^ on an Mettler-Toledo TGA/DSC 3+ instrument. Approximately 2 mg of the sample were placed in a standard alumina crucible (70 µl).

## Synthesis and crystallization   

6-Fluoro­nicotinic acid (0.0495 g, 0.3508 mmol) was dissolved in distilled water (5 ml), 4,4′-bi­pyridine (0.0276 g, 0.1767 mmol) was dissolved in ethanol (2 mL) and nickel(II) sulfate hepta­hydrate (0.0517 g, 0.1841 mmol) was dissolved in distilled water (2 mL). The solutions of the two ligands were first mixed together under stirring. The resulting solution was then slowly added to the nickel(II) sulfate solution under stirring. The pH of the final solution was adjusted to 7 by adding an ammonia solution dropwise. The obtained, clear solution was left to evaporate slowly at room temperature for approximately three weeks until light–blue crystals of **1**, suitable for X-ray diffraction measurements, were obtained. These were collected by filtration, washed with their mother liquor and dried *in vacuo*. Yield: 0.0483 g (45%). Selected IR bands (ATR) (*ν*, cm^−1^): 3351 [*ν*(O—H)], 3088 [ν(C—H)], 1607 [ν(C=O)], 1558, 1475, 1415, 1392, 1368 [ν(C—C), ν(C—N)] (see Fig. S2, Table S1 in the supporting information).

## Refinement   

Crystal data, data collection and structure refinement details are summarized in Table 2 ▸. C-bound atoms were positioned geometrically and refined using a riding model [0.93 Å, *U*_iso_(H) = 1.2*U*_eq_(C) for aromatic H atoms]. Water H atoms were found in difference-Fourier maps, O—H distances were restrained to an average value of 0.82 Å using DFIX and DANG instructions and they were refined isotropically [*U*_iso_(H) = 1.2*U*_eq_(O)].

The highest difference peak is 0.92 Å away from the O3 atom and the deepest difference hole is 0.50 Å away from the Ni1 atom.

## Supplementary Material

Crystal structure: contains datablock(s) I. DOI: 10.1107/S2056989020003023/xi2024sup1.cif

Structure factors: contains datablock(s) I. DOI: 10.1107/S2056989020003023/xi2024Isup4.hkl

TGA, DSC and IR data. DOI: 10.1107/S2056989020003023/xi2024sup3.docx

CCDC reference: 1988000

Additional supporting information:  crystallographic information; 3D view; checkCIF report

## Figures and Tables

**Table 1:** Hydrogen-bond geometry (Å, °)

*D*—H⋯*A*	*D*—H	H⋯*A*	*D*⋯*A*	*D*—H⋯*A*
O1—H12⋯O4	0.82 (1)	2.05 (1)	2.848 (2)	166 (2)
O1—H11⋯O3^i^	0.82 (1)	1.88 (1)	2.674 (2)	163 (2)
O4—H41⋯O5^ii^	0.82 (1)	1.99 (1)	2.811 (3)	175 (3)
O5—H51⋯O3^iii^	0.82 (1)	2.24 (3)	2.964 (4)	147 (4)
O5—H52⋯N1^iv^	0.82 (1)	2.41 (3)	3.100 (3)	142 (4)

Symmetry codes: (i) 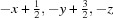; (ii) 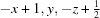; (iii) 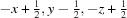; (iv) 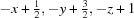.

**Table 2:** Experimental details

Crystal data	
Chemical formula	{[Ni(C_6_H_3_FNO_2_)_2_(C_10_H_8_N_2_)(H_2_O)_2_]·3H_2_O}_*n*_
*M*_r_	585.16
Crystal system, space group	Monoclinic, *C*2/*c*
Temperature (K)	296
*a*, *b*, *c* (Å)	12.1175 (5), 18.7705 (6), 12.3246 (4)
β (°)	110.232 (4)
*V* (Å^3^)	2630.29 (17)
*Z*	4
Radiation type	Mo *K*α
μ (mm^−1^)	0.81
Crystal size (mm)	0.15 × 0.10 × 0.08

Data collection	
Diffractometer	Oxford Diffraction Xcalibur2 diffractometer with Sapphire 3 CCD detector
Absorption correction	Multi-scan (*CrysAlis PRO*; Rigaku, 2018 ▸)
*T*_min_, *T*_max_	0.899, 1.000
No. of measured, independent and observed [*I* > 2σ(*I*)] reflections	5540, 2316, 1960
*R*_int_	0.032
(sin θ/λ)_max_ (Å^−1^)	0.595

Refinement	
*R*[*F* ^2^ > 2σ(*F* ^2^)], *wR*(*F* ^2^), *S*	0.032, 0.073, 1.03
No. of reflections	2316
No. of parameters	189
No. of restraints	7
H-atom treatment	H atoms treated by a mixture of independent and constrained refinement
Δρ_max_, Δρ_min_ (e Å^−3^)	0.30, −0.22

Computer programs: *CrysAlis PRO* (Rigaku, 2018 ▸), *SHELXT* (Sheldrick, 2015*a* ▸), *SHELXL2018/3* (Sheldrick, 2015*b* ▸) and *Mercury* (Macrae *et al.*, 2020 ▸).

## supplementary crystallographic information

### Crystal data

**Table:**

[Ni(C_6_H_3_FNO_2_)_2_(C_10_H_8_N_2_)(H_2_O)_2_]·3H_2_O	*F*(000) = 1208
*M**_r_* = 585.16	*D*_x_ = 1.478 Mg m^−^^3^
Monoclinic, *C*2/*c*	Mo *K*α radiation, λ = 0.71073 Å
*a* = 12.1175 (5) Å	Cell parameters from 2838 reflections
*b* = 18.7705 (6) Å	θ = 4.6–31.4°
*c* = 12.3246 (4) Å	µ = 0.81 mm^−^^1^
β = 110.232 (4)°	*T* = 296 K
*V* = 2630.29 (17) Å^3^	Prism, light-blue
*Z* = 4	0.15 × 0.10 × 0.08 mm

### Data collection

**Table:**

Oxford Diffraction Xcalibur2 diffractometer with Sapphire 3 CCD detector	1960 reflections with *I* > 2σ(*I*)
ω–scan	*R*_int_ = 0.032
Absorption correction: multi-scan (CrysAlisPro; Rigaku, 2018)	θ_max_ = 25.0°, θ_min_ = 4.1°
*T*_min_ = 0.899, *T*_max_ = 1.000	*h* = −13→14
5540 measured reflections	*k* = −22→16
2316 independent reflections	*l* = −14→12

### Refinement

**Table:**

Refinement on *F*^2^	7 restraints
Least-squares matrix: full	Hydrogen site location: mixed
*R*[*F*^2^ > 2σ(*F*^2^)] = 0.032	H atoms treated by a mixture of independent and constrained refinement
*wR*(*F*^2^) = 0.073	*w* = 1/[σ^2^(*F*_o_^2^) + (0.033*P*)^2^ + 1.7768*P*] where *P* = (*F*_o_^2^ + 2*F*_c_^2^)/3
*S* = 1.02	(Δ/σ)_max_ < 0.001
2316 reflections	Δρ_max_ = 0.30 e Å^−^^3^
189 parameters	Δρ_min_ = −0.22 e Å^−^^3^

### Special details

**Table:**

Geometry. All esds (except the esd in the dihedral angle between two l.s. planes) are estimated using the full covariance matrix. The cell esds are taken into account individually in the estimation of esds in distances, angles and torsion angles; correlations between esds in cell parameters are only used when they are defined by crystal symmetry. An approximate (isotropic) treatment of cell esds is used for estimating esds involving l.s. planes.

### Fractional atomic coordinates and isotropic or equivalent isotropic displacement parameters (Å^2^)

**Table:**

	*x*	*y*	*z*	*U*_iso_*/*U*_eq_	
Ni1	0.250000	0.750000	0.000000	0.02551 (12)	
N1	0.2304 (2)	0.89116 (14)	0.49414 (19)	0.0627 (7)	
N2	0.16406 (15)	0.65554 (9)	0.00035 (13)	0.0283 (4)	
O1	0.41435 (14)	0.69897 (8)	0.06960 (13)	0.0365 (4)	
H12	0.429 (2)	0.6719 (11)	0.1244 (14)	0.044*	
H11	0.416 (2)	0.6772 (11)	0.0127 (14)	0.044*	
O2	0.25264 (13)	0.77061 (8)	0.16466 (12)	0.0349 (4)	
O3	0.12044 (17)	0.85740 (11)	0.13890 (14)	0.0661 (6)	
O4	0.500000	0.59666 (15)	0.250000	0.0563 (7)	
H41	0.539 (2)	0.5710 (13)	0.223 (3)	0.068*	
O5	0.3692 (3)	0.51516 (15)	0.3540 (2)	0.1055 (9)	
H51	0.401 (4)	0.4760 (12)	0.372 (3)	0.127*	
H52	0.374 (4)	0.533 (2)	0.416 (2)	0.127*	
F1	0.35722 (18)	0.86788 (12)	0.67067 (13)	0.0934 (6)	
C1	0.1919 (3)	0.87822 (15)	0.3797 (2)	0.0542 (7)	
H1	0.128197	0.904258	0.332209	0.065*	
C2	0.2426 (2)	0.82806 (12)	0.32961 (19)	0.0385 (5)	
C3	0.3359 (2)	0.78930 (14)	0.4018 (2)	0.0438 (6)	
H3	0.371596	0.754740	0.371012	0.053*	
C4	0.3763 (2)	0.80150 (16)	0.5186 (2)	0.0527 (7)	
H4	0.438816	0.776057	0.569207	0.063*	
C5	0.3193 (3)	0.85305 (17)	0.5559 (2)	0.0600 (8)	
C6	0.2003 (2)	0.81802 (12)	0.20071 (19)	0.0373 (5)	
C7	0.1834 (2)	0.61732 (12)	0.09598 (18)	0.0420 (6)	
H7	0.240902	0.632605	0.163889	0.050*	
C8	0.1220 (2)	0.55619 (12)	0.09870 (19)	0.0452 (6)	
H8	0.138908	0.530975	0.167511	0.054*	
C9	0.03574 (17)	0.53205 (10)	0.00029 (16)	0.0266 (4)	
C10	0.0173 (2)	0.57183 (12)	−0.09902 (18)	0.0377 (5)	
H10	−0.039219	0.557751	−0.168379	0.045*	
C11	0.0828 (2)	0.63211 (12)	−0.09476 (17)	0.0375 (5)	
H11A	0.069114	0.657839	−0.162659	0.045*	

### Atomic displacement parameters (Å^2^)

**Table:**

	*U*^11^	*U*^22^	*U*^33^	*U*^12^	*U*^13^	*U*^23^
Ni1	0.0328 (2)	0.0207 (2)	0.02366 (19)	−0.00983 (16)	0.01060 (15)	−0.00195 (14)
N1	0.0853 (19)	0.0660 (16)	0.0442 (13)	−0.0026 (14)	0.0320 (14)	−0.0202 (11)
N2	0.0347 (9)	0.0245 (9)	0.0257 (9)	−0.0092 (7)	0.0103 (8)	−0.0014 (7)
O1	0.0415 (9)	0.0348 (9)	0.0312 (8)	−0.0032 (7)	0.0101 (7)	0.0016 (6)
O2	0.0493 (9)	0.0301 (8)	0.0292 (8)	−0.0061 (7)	0.0183 (7)	−0.0036 (6)
O3	0.0746 (13)	0.0801 (14)	0.0386 (10)	0.0310 (12)	0.0133 (10)	−0.0040 (9)
O4	0.0750 (19)	0.0426 (16)	0.0524 (16)	0.000	0.0233 (14)	0.000
O5	0.173 (3)	0.0724 (18)	0.101 (2)	0.0023 (18)	0.085 (2)	0.0076 (15)
F1	0.1125 (15)	0.1310 (18)	0.0359 (9)	−0.0144 (13)	0.0247 (10)	−0.0284 (10)
C1	0.0651 (17)	0.0574 (17)	0.0441 (14)	0.0054 (14)	0.0239 (13)	−0.0079 (12)
C2	0.0444 (13)	0.0405 (14)	0.0350 (12)	−0.0078 (11)	0.0194 (11)	−0.0058 (10)
C3	0.0456 (13)	0.0506 (16)	0.0393 (13)	−0.0050 (12)	0.0200 (11)	−0.0038 (11)
C4	0.0487 (15)	0.072 (2)	0.0356 (13)	−0.0083 (13)	0.0128 (12)	0.0001 (12)
C5	0.073 (2)	0.078 (2)	0.0317 (14)	−0.0204 (17)	0.0214 (14)	−0.0136 (13)
C6	0.0427 (13)	0.0384 (13)	0.0325 (12)	−0.0056 (11)	0.0153 (11)	−0.0033 (10)
C7	0.0527 (14)	0.0393 (14)	0.0252 (11)	−0.0246 (11)	0.0022 (10)	0.0008 (9)
C8	0.0619 (15)	0.0379 (14)	0.0273 (11)	−0.0245 (12)	0.0047 (11)	0.0071 (10)
C9	0.0331 (10)	0.0226 (11)	0.0243 (10)	−0.0065 (8)	0.0103 (9)	−0.0025 (8)
C10	0.0444 (13)	0.0368 (13)	0.0267 (11)	−0.0196 (10)	0.0057 (10)	−0.0013 (9)
C11	0.0478 (13)	0.0351 (13)	0.0256 (11)	−0.0181 (11)	0.0074 (10)	0.0051 (9)

### Geometric parameters (Å, º)

**Table:**

Ni1—O2^i^	2.0553 (13)	F1—C5	1.357 (3)
Ni1—O2	2.0553 (13)	C1—C2	1.381 (3)
Ni1—N2^i^	2.0570 (16)	C1—H1	0.9300
Ni1—N2	2.0570 (16)	C2—C3	1.380 (3)
Ni1—O1^i^	2.1067 (16)	C2—C6	1.503 (3)
Ni1—O1	2.1067 (16)	C3—C4	1.370 (3)
N1—C5	1.298 (4)	C3—H3	0.9300
N1—C1	1.345 (3)	C4—C5	1.358 (4)
N2—C11	1.319 (3)	C4—H4	0.9300
N2—C7	1.330 (3)	C7—C8	1.374 (3)
O1—H12	0.815 (10)	C7—H7	0.9300
O1—H11	0.819 (9)	C8—C9	1.376 (3)
O2—C6	1.260 (3)	C8—H8	0.9300
O3—C6	1.246 (3)	C9—C10	1.384 (3)
O4—H41	0.822 (10)	C9—C9^iii^	1.481 (4)
O4—H41^ii^	0.822 (10)	C10—C11	1.373 (3)
O5—H51	0.823 (10)	C10—H10	0.9300
O5—H52	0.820 (10)	C11—H11A	0.9300
			
O2^i^—Ni1—O2	180.00 (8)	C3—C2—C6	121.3 (2)
O2^i^—Ni1—N2^i^	89.73 (6)	C1—C2—C6	121.1 (2)
O2—Ni1—N2^i^	90.27 (6)	C4—C3—C2	120.3 (2)
O2^i^—Ni1—N2	90.27 (6)	C4—C3—H3	119.9
O2—Ni1—N2	89.73 (6)	C2—C3—H3	119.9
N2^i^—Ni1—N2	180.0	C5—C4—C3	115.9 (3)
O2^i^—Ni1—O1^i^	89.65 (6)	C5—C4—H4	122.1
O2—Ni1—O1^i^	90.35 (6)	C3—C4—H4	122.1
N2^i^—Ni1—O1^i^	90.87 (6)	N1—C5—F1	114.3 (3)
N2—Ni1—O1^i^	89.13 (6)	N1—C5—C4	127.6 (2)
O2^i^—Ni1—O1	90.35 (6)	F1—C5—C4	118.1 (3)
O2—Ni1—O1	89.65 (6)	O3—C6—O2	125.6 (2)
N2^i^—Ni1—O1	89.13 (6)	O3—C6—C2	118.7 (2)
N2—Ni1—O1	90.87 (6)	O2—C6—C2	115.6 (2)
O1^i^—Ni1—O1	180.0	N2—C7—C8	122.8 (2)
C5—N1—C1	115.6 (2)	N2—C7—H7	118.6
C11—N2—C7	117.17 (18)	C8—C7—H7	118.6
C11—N2—Ni1	120.57 (13)	C7—C8—C9	120.4 (2)
C7—N2—Ni1	122.19 (14)	C7—C8—H8	119.8
Ni1—O1—H12	122.0 (17)	C9—C8—H8	119.8
Ni1—O1—H11	100.5 (17)	C8—C9—C10	116.28 (18)
H12—O1—H11	110 (2)	C8—C9—C9^iii^	122.5 (2)
C6—O2—Ni1	130.05 (14)	C10—C9—C9^iii^	121.2 (2)
H41—O4—H41^ii^	108 (4)	C11—C10—C9	119.77 (19)
H51—O5—H52	103 (3)	C11—C10—H10	120.1
N1—C1—C2	123.0 (3)	C9—C10—H10	120.1
N1—C1—H1	118.5	N2—C11—C10	123.57 (19)
C2—C1—H1	118.5	N2—C11—H11A	118.2
C3—C2—C1	117.6 (2)	C10—C11—H11A	118.2
			
C5—N1—C1—C2	−0.2 (4)	C1—C2—C6—O3	2.1 (3)
N1—C1—C2—C3	0.9 (4)	C3—C2—C6—O2	2.5 (3)
N1—C1—C2—C6	−177.0 (2)	C1—C2—C6—O2	−179.8 (2)
C1—C2—C3—C4	−0.7 (4)	C11—N2—C7—C8	−0.6 (4)
C6—C2—C3—C4	177.2 (2)	Ni1—N2—C7—C8	176.35 (19)
C2—C3—C4—C5	−0.2 (4)	N2—C7—C8—C9	−0.4 (4)
C1—N1—C5—F1	179.2 (2)	C7—C8—C9—C10	1.1 (3)
C1—N1—C5—C4	−0.9 (4)	C7—C8—C9—C9^iii^	−177.8 (3)
C3—C4—C5—N1	1.1 (4)	C8—C9—C10—C11	−0.8 (3)
C3—C4—C5—F1	−179.0 (2)	C9^iii^—C9—C10—C11	178.1 (2)
Ni1—O2—C6—O3	11.0 (3)	C7—N2—C11—C10	1.0 (3)
Ni1—O2—C6—C2	−166.92 (13)	Ni1—N2—C11—C10	−176.03 (18)
C3—C2—C6—O3	−175.6 (2)	C9—C10—C11—N2	−0.3 (4)

Symmetry codes: (i) −*x*+1/2, −*y*+3/2, −*z*; (ii) −*x*+1, *y*, −*z*+1/2; (iii) −*x*, −*y*+1, −*z*.

### Hydrogen-bond geometry (Å, º)

**Table:**

*D*—H···*A*	*D*—H	H···*A*	*D*···*A*	*D*—H···*A*
O1—H12···O4	0.82 (1)	2.05 (1)	2.848 (2)	166 (2)
O1—H11···O3^i^	0.82 (1)	1.88 (1)	2.674 (2)	163 (2)
O4—H41···O5^ii^	0.82 (1)	1.99 (1)	2.811 (3)	175 (3)
O5—H51···O3^iv^	0.82 (1)	2.24 (3)	2.964 (4)	147 (4)
O5—H52···N1^v^	0.82 (1)	2.41 (3)	3.100 (3)	142 (4)
C11—H11*A*···O2^i^	0.93	2.55	3.040 (2)	113

Symmetry codes: (i) −*x*+1/2, −*y*+3/2, −*z*; (ii) −*x*+1, *y*, −*z*+1/2; (iv) −*x*+1/2, *y*−1/2, −*z*+1/2; (v) −*x*+1/2, −*y*+3/2, −*z*+1.
