# Supplementary material for: The first coordination compound of 6-fluoro­nicotinate: the crystal structure of a one-dimensional nickel(II) coordination polymer containing the mixed ligands 6-fluoro­nicotinate and 4,4′-bi­pyridine
Source: Acta Crystallogr E Crystallogr Commun. 2020 Mar 10;76(Pt 4):500–5. doi: 10.1107/S2056989020003023 (PMC7133043; doi:10.1107/S2056989020003023)
Supplement: Supplementary file 3 [file e-76-00500-sup3.docx]

**The first coordination compound of 6-fluoronicotinate: crystal structure of a nickel(II) one-dimensional coordination polymer containing mixed ligands 6-fluoronicotinate and 4,4'-bipyridine**

**Nives Politeo,**^a^ **Mateja Pisa**č**i**ć**,**^b^ **Marijana** Đ**akovi**ć**,**^b^ **Vesna Sokol**^a^*** and Boris-Marko Kukovec**^a^

**^a^**Department of Physical Chemistry, Faculty of Chemistry and Technology, University of Split, Ruđera Boškovića 35, HR-21000 Split, Croatia, and **^b^**Department of Chemistry, Faculty of Science, University of Zagreb, Horvatovac, 102a, HR-10000 Zagreb, Croatia

Correspondence email: vsokol@ktf-split.hr

**Click the grey 'Authors' label above or use the 'IUCr authors' toolbar button to edit the authors**

Supporting information


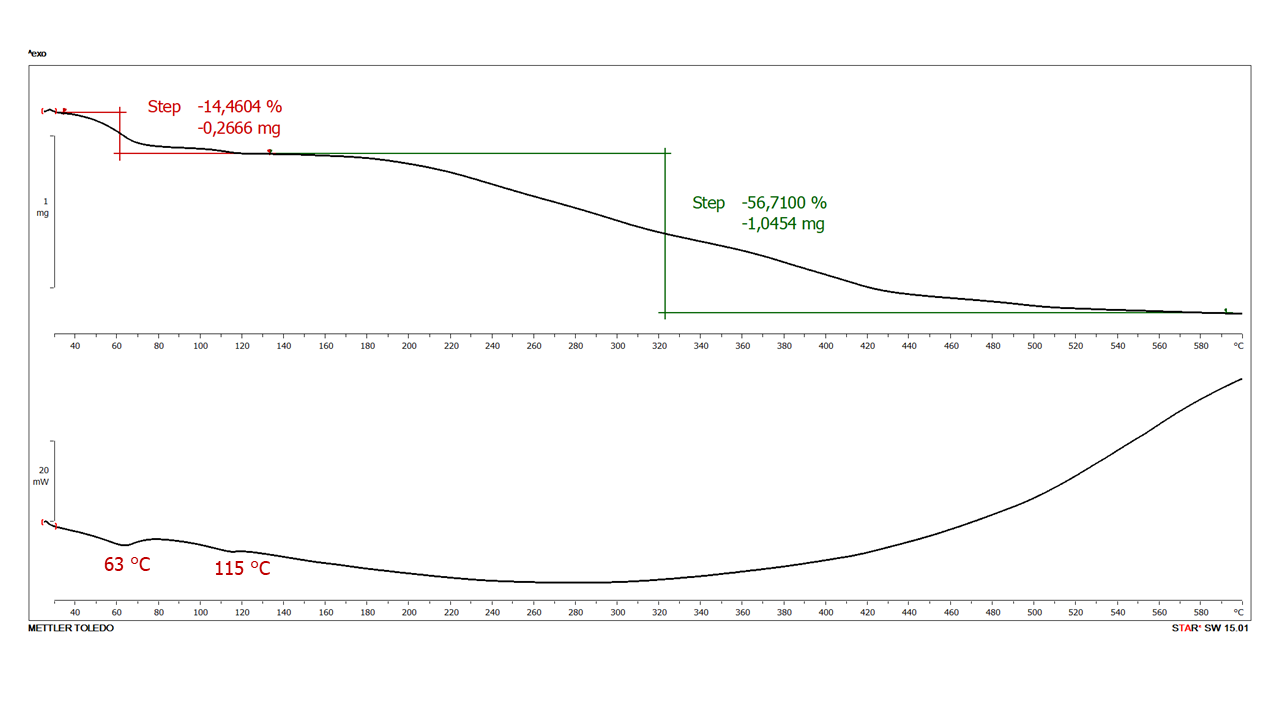


1. TGA (top) and DSC (bottom) curves of **1**.


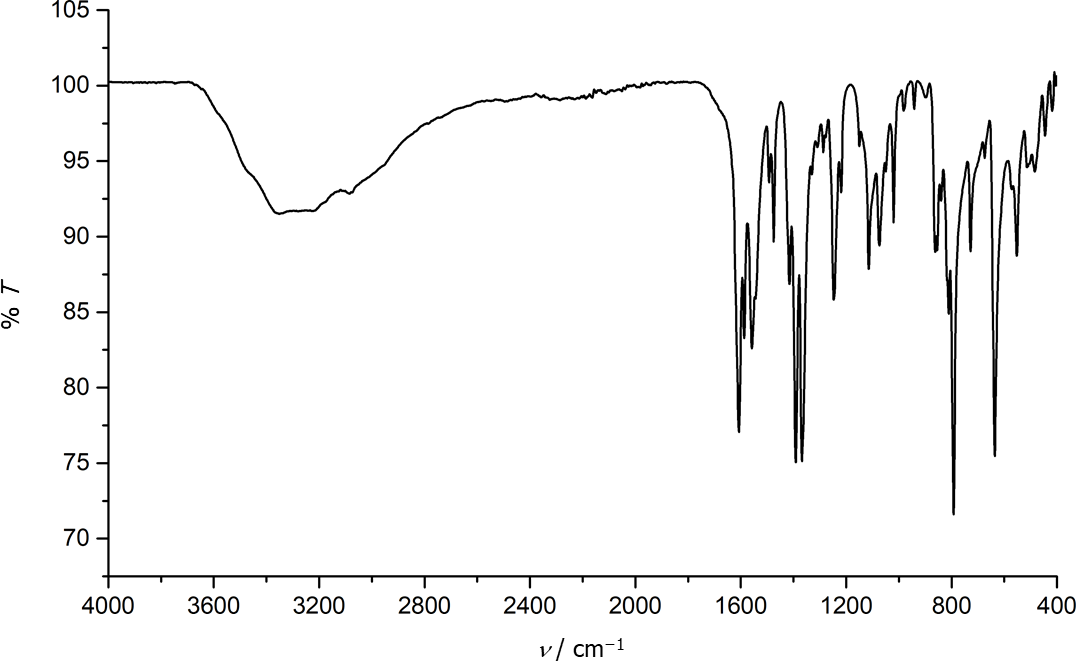


1. IR spectrum of **1**.

**Table S1** IR bands of **1**.

| *ν* (cm^−1^) | %*T* |
| --- | --- |
| 3351 | 91 |
| 3218 | 92 |
| 3088 | 93 |
| 1607 | 77 |
| 1587 | 83 |
| 1558 | 82 |
| 1475 | 89 |
| 1415 | 86 |
| 1392 | 74 |
| 1368 | 74 |
| 1247 | 84 |
| 1219 | 91 |
| 1115 | 86 |
| 1074 | 87 |
| 1020 | 88 |
| 855 | 85 |
| 811 | 80 |
| 792 | 67 |
| 728 | 82 |
| 636 | 68 |
